# Endoparasites infecting exotic captive amphibian pet and zoo animals (Anura, Caudata) in Germany

**DOI:** 10.1007/s00436-020-06876-0

**Published:** 2020-09-22

**Authors:** Malek J. Hallinger, Anja Taubert, Carlos Hermosilla

**Affiliations:** 1 Institute of Parasitology, Justus Liebig University Giessen, Schubertstr. 81, Biomedical Research Centre Seltersberg, 35392 Giessen, Germany; 2 exomed GmbH, Schönhauser Str. 62, 13127 Berlin, Germany

**Keywords:** Amphibians, Parasites, Caudata, Anura, Endoparasites, Exotic pets, Exotic pet medicine

## Abstract

Alongside exotic reptiles, amphibians, such as toads, frogs, salamanders, and newts, are nowadays considered popular pets worldwide. As reported for other exotic pet animals, amphibians are known to harbor numerous gastrointestinal parasites. Nonetheless, very little data are available on captive amphibian parasitic diseases. In this study, we applied direct saline fecal smears (DSFS) to examine in total 161 stool samples from 41 different amphibian species belonging to the orders Anura and Caudata. In addition, carbolfuchsin-smear (CFS) staining (*n* = 74 samples) was used to detect amphibian *Cryptosporidium* oocysts. Also, complete dissections of deceased amphibians (*n* = 107) were performed to specify parasite infections and to address parasite-associated pathogenicity. Overall, examined amphibian fecal samples contained 12 different parasite taxa. The order Rhabditida with the species *Rhabdias* spp. and *Strongyloides* spp. were the most prevalent nematode species (19.3%), followed by flagellated protozoans (8.7%), *Amphibiocapillaria* spp./*Neocapillaria* spp. (7.5%), *Oswaldocruzia* spp. (4.3%), *Blastocystis* spp. (3.1%), *Cosmocerca* spp. (3.1%), oxyurids (Pharyngonoidae) (3.1%), spirurids (1.2%), un-sporulated coccidian oocysts (0.6%), *Tritrichomonas* spp. (0.6%), *Karotomorpha* spp. (0.6%), and *Cryptosporidium* spp. (0.6%). One CFS-stained fecal sample (1.4%) was positive for *Cryptosporidium* oocysts. Within dissected amphibians, 31 (48.4%) of the anurans and 11 (26.2%) of the salamanders were infected with gastrointestinal parasites. One cutaneous *Pseudocapillaroides xenopi* infection was diagnosed in an adult African clawed frog (*Xenopus laevis*). Etiologically, 17 (15.9%) of them died due to severe parasitic and/or bacterial infections (e.g., *Chryseobacterium indologenes*, *Citrobacter freudii*, *Sphingobacterium multivorum*, *Klebsiella pneumoniae*). High prevalence and pathological findings of several clinical amphibian parasitoses call for more detailed investigation on gastrointestinal parasite-derived molecular mechanisms associated with detrimental lesions or even death.

## Introduction

Amphibian species are ectothermic, tetrapod, and mainly carnivorous vertebrate species of the class Amphibia (Hill et al. 2015). Modern amphibians inhabit a wide variety of habitats, with most species living within terrestrial, fossorial, arboreal, or freshwater aquatic ecosystems. In the last decades, amphibians established themselves as domestic exotic pets/zoo animals worldwide (Mutschmann 2010). Amphibian species have suffered a significant decline in the wild during the last decades, mainly due to anthropogenic pressure, such as environmental contamination, UV-B irradiation, introduction of alien/invasive species, direct mistreatment, habitat losses, climate changes, and emerging diseases (e.g., chytridiomycosis, Ranavirus) (Daszak et al. 1999; Stuart et al. 2004; Beebee and Griffiths 2005; Collins 2010; Bishop et al. 2012; Henle et al. 2012; Foden et al. 2013; Martel et al. 2014; Wren et al. 2015; Nguyen et al. 2017) and have attracted special media and public attention. This public attention among other factors has raised interest for these ectothermic animals resulting in an increased popularity of amphibians as private pets, also in Germany (Krautwald-Junghanns 2017).

International amphibian trade is becoming governmentally more and more restricted in many countries. Radical restrictions seem to be impossible to implement and also require time, effort, and knowledge with no guarantee of success. Even non-regulated trade/black market activities happen as suggested elsewhere (Garner et al. 2009; Bishop et al. 2012). More importantly, diverse anuran (i.e., frogs and toads) and caudate (i.e., salamanders and newts) amphibians threatened by extinction are nowadays part of many zoological collections worldwide (Bishop et al. 2012; Ziegler 2016; Ziegler and Rauhaus 2019).

Since there is a considerable overlap within described diseases for captive amphibians/reptiles/fishes and free-ranging amphibians, caretakers must be mindful on endoparasites and diseases while co-housing amphibians with wild ones or other zoological taxa, such as fishes (Densmore and Green 2007). Parasitic diseases of amphibians are closely related to parasitoses affecting other ectothermic vertebrates (Densmore and Green 2007). Therefore, parasite species can be transmitted from other ectothermic vertebrates to amphibians (i.e., fish ectoparasites, such as the protozoan species, *Trichodina* spp. and *Ichthyobodo* spp.) (Densmore and Green 2007; Mutschmann 2010). Most amphibian pets or other lower vertebrates, such as reptiles maintained in captivity, are often associated to inadequate husbandry and mismanagement conditions (Beck and Pantchev 2013; Wolf et al. 2014). In addition, specific intrinsic associated risk factors (e.g., age, sex, species, host immune status) and extrinsic risk factors (e.g., poor hygiene housing conditions, temperature, humidity, animal density, nutrition) might lead to relevant parasitic burdens (Mutschmann 2010; Beck and Pantchev 2013; Hallinger et al. 2019; 2020). Reinfection with resistant reproductive stages of certain endoparasites (e.g., oxyurid eggs) can lead to heavy parasitism and/or even death of pet reptiles/amphibians (Frank 1981; Pasmans 2008; Beck and Pantchev 2013; Wolf et al. 2014; Hallinger et al. 2018).

Amphibian hosts can be infected with different gastrointestinal parasites, such as protozoans, nematodes, cestodes, trematodes, acanthocephalans, and pentastomids (Frank 1984; Vaucher 1990; Al-Sorakhy and Amr 2003; Barton and Riley 2004; Densmore and Green 2007; Mutschmann 2010). Some of them bear zoonotic potential, such as helminths (*Spirometra* spp., *Gnathostoma* spp., *Diphyllobothrium* spp., *Alaria* spp., and *Echinostoma* spp.) and pentastomids (Pentastoma), since most of them are food-borne diseases (Graczyk und Fried 1998; de Górgolas et al. 2003; Dorny et al. 2009; Pantchev und Tappe 2011; Warwick et al. 2012). Up to date, most scientific research have focused on free-ranging amphibians or laboratory animals (Coggins and Sajdak 1982; Cunningham et al. 1996; Hamann et al. 2012; Kuzmin et al. 2003; Loras et al. 2010; Mohammad et al. 2010; Rizvi and Bhutia 2010; Amin et al. 2012; Yildirimhan et al. 2012), but seldom on captivity kept anuran and caudate amphibians. Thus, this comprehensive investigation on German captive amphibian pets of private households and German zoological collections aims to provide current data on the occurrence of gastrointestinal endoparasites, to assess differences in parasite occurrence between privately kept animals and zoo animals considering host species, keeping facility, sex, and/or order/taxon. In addition, we aim to assess presence of zoonotic parasites circulating in amphibian pets and further to gain a better understanding of parasite-derived pathogenicity in these exotic herpetic pets.

## Materials and methods

### Fecal samples

Examined fecal samples originated either from animals owned privately, submitted by owners attending veterinarians, or by different German zoos entities, which had been referred to exomed® laboratory in Berlin, Germany. In order to identify both protozoan and helminth stages, we performed direct saline fecal smears (DSFS) for general parasitological diagnosis according to Barnard and Upton (1994). Clients were also asked to provide a printed form containing individual animal’s signalement (i.e., species, sex, age), husbandry circumstances (i.e., origin, animal density, time in owner’s possession), previous parasitological examinations, and anthelminthic treatments. At exomed® laboratory, all stool samples were labeled with corresponding forms and reference numbers and finally conserved at 4 °C for up to 2 h in a lab refrigerator until further parasitological examination.

For DSFS, a uniform solution was created by mixing 1 g of amphibian feces at a ratio of 1:1 with 0.9% saline solution, carefully placed on glass cover slides (Nunc) with pipette (Nunc) and finally covered with cover slips (22 × 22 mm; Nunc). Both a 100× and/or 400× magnification for light microscopy examination (Axio Imager M1®, Zeiss, Jena) equipped with a digital camera were used here.

Consistent, metazoan parasitic stages (i.e., eggs, proglottids, larvae, nematodes) and protozoan parasitic stages (i.e., trophozoites, cysts and oocyst) were identified based on previous morphological/-metric descriptions as reported elsewhere (Frank 1984, 1985; Mutschmann 2010). Samples were classified as “positive” when at least one stage of an endoparasite was found in fecal smears (Table 1). Samples containing apathogenic flagellates/ciliates (e.g., *Nyctotherus*) or opalozoans (e.g., *Opalina* spp., *Protoopalina* spp.) were classified as “negative” according to previous reports (Corliss 1955; Frank 1984, 1985; Densmore and Green 2007; Mutschmann 2010). Additionally, samples were analyzed by carbolfuchsin-stained (CFS) smears for detection of *Cryptosporidium* oocysts.

**Table 1:** Examined fecal samples of amphibians and origin of sender (total *n* = 161) regarding infection rate with endoparasites (%)

Amphibian order (number of different examined species)	Common name	No. examined	Origin (private/vet/zoo)	Positive for endoparasites (%)
Anura (37)	Frogs/toads	127	106/13/8	58 (45.7)
Caudata (6)	Salamanders	32	24/6/2	6 (9.4)
unknown (2)	Unknown	2	2/0/0	0 (0.0)

### Amphibian autopsies

Amphibian corpses of deceased pet animals were necropsied at exomed® laboratory (Table 2, Online supplement). Clients were also asked to provide a printed form containing individual animal’s signalement (i.e., species, sex, age) and husbandry circumstances (i.e., origin, animal density, time in owner’s possession). Additionally, amphibian hosts were morphologically identified using corresponding published literature (Hofrichter 2000). In addition, pathohistological examinations were performed using standard hematoxylin and eosin (H&E) batch staining (Buesa 2007) of the following amphibian organs: liver, lungs, intestine, and kidneys. First, a visual inspection of the whole digestive tract was conducted to unveil presence of macroscopic helminth endoparasites. Afterwards, intestinal contents were examined by DSFS method. Morphological identification of endoparasites was performed under a light microscope equipped with a digital camera (Axio Vision M1®, Zeiss, Jena). External examinations and necropsies were performed as described previously by Mutschmann (2010).

**Table 2:** Performed necropsies of amphibians, order, and origin of sender (*n* = 107) regarding infection rate with endoparasites (%)

Amphibian order (number of examined species)	Common name	No. examined	Origin (private/vet/zoo)	Positive for endoparasites (%)
Anura (25)	Frogs/toads	64	25/10/29	31 (48.4)
Caudata (16)	Salamanders	42	22/6/14	11 (26.2)
Gymnophiona (1)	Caecillians	1	0/0/1	0 (0.0)

### Microbiology

If requested, feces or coelom swabs were inoculated on different agar plates for bacterial and fungal cultivation. As such, further pathogen isolation on sheep blood agars (5%), MacConkey agars, alongside Sabourand dextrose agars (SDA) (BioMerieux, Charbonnier les Bains, France) was performed. Bacterial isolates were diagnosed by Gram straining, oxidase and catalase tests, as well as a commercially available API 20E/NE® kit (BioMerieux, Charbonnier les Bains, France) as described for poikilothermal vertebrates, such as amphibians and reptiles (Marenzoni et al. 2015; Hallinger et al. 2018).

## Results

From October 2015 to January 2019, coprological analyses of 161 amphibian fecal samples were performed (Table 3). These scat samples originated from 41 different amphibian species enabling to generate representative prevalence data (Online Supplement). In these samples, we recorded 12 different parasite species (please refer to Table 4). *Rhabdias* and *Strongyloides* (Rhabditida) were the most prevalent metazoan parasitic genera (19.3%). Furthermore, 14 samples (8.7%) contained flagellated protozoans (Metamonada) and 12 samples *Amphibiocapillaria/Neocapillaria* spp. (7.5%), 7 samples *Oswaldocruzia* spp. (4.3%), 5 samples *Blastocystis* spp. (3.1%), 5 samples *Cosmocerca* spp. (3.1%), 4 samples oxyurids (Pharyngonoidae) (2.5%), two samples spirurids (1.2%), and one sample un-sporulated coccidian oocysts (0.6%). In addition, *Tritrichomonas* trophozoites (0.6%), *Karotomorpha* trophozoites (0.6%), and *Cryptosporidium* oocysts (0.6%) were present in the fecal samples (*n* = 73).

**Table 3:** Examined fecal samples of amphibians (total *n* = 161)

Order/species	Common name	Author/year of description	Examined fecal samples (*n* = 161)
Anura			127
*Litoria caerulea*	Green tree frog	White, 1790	31
*Dendrobates tinctorius*	Dyeing dart frog	Cuvier, 1797	21
*Agalychnis callidryas*	Red-eyed treefrog	Cope, 1862	12
*Ceratophrys cranwelli*	Chacoan horned frog	Barrio, 1980	8
*Dendrobates* sp.	–	–	7
*Dendrobates auratus*	Green-and-black poison dart frog	Girard, 1855	3
*Oophaga pumilio*	Strawberry poison frog	Schmidt, 1857	3
*Ceratophrys ornata*	Argentine horned frog	Bell, 1843	2
*Pyxicephalus adspersus*	African bullfrog	Tschudi, 1838	2
*Ranitomeya ventrimaculata*	Reticulated poison frog	Shreve, 1935	2
*Epipedobates anthonyi*	Anthony’s poison arrow frog	Noble, 1921	3
*Xenopus laevis*	African clawed frog	Daudin 1802	2
*Oophaga histrionica*	Harlequin poison frog	Berthold, 1845	1
*Phyllobates vitatus*	Golfodulcean poison frog	Cope, 1893	1
*Phyllobates bicolor*	Black-legged poison frog	Duméril and Bibron, 1841	1
*Bufo regularis*	African common toad	Reuss, 1833	1
*Ranitomeya imitator*	Mimic poison frog	Schulte, 1986	1
*Agalychnis spurelli*	Gliding leaf frog	Boulenger, 1913	1
*Osornophryne guacamayo*	Guacamayo plump toad	Hoogmoed, 1987	1
*Sycirax wampukrum*	–	Bravo, 2009	1
*Phyllobates terribilis*	Golden poison frog	Myers, Daly, and Malkin, 1978	4
*Adelphobates galactonotus*	Splash-backed poison frog	Steindachner, 1864	2
*Phyllomedusa bicolor*	Blue-and-yellow frog	Boddaert, 1772	1
*Megophrys nasuta*	Long-nosed horned frog	Schlegel, 1858	1
*Anaxyrus debilis*	North American green toad	Girard, 1854	1
*Melanophryniscus klappenbachi*	Klappenbach’s red-bellied frog	Prigioni and Langone, 2000	1
*Trachycephalus resinifictrix*	Amazon milk frog	Goeldi, 1907	2
*Bombina microdeladigitora*	Guangxi firebelly toad	Tian and Wu, 1978	1
*Bombina orientalis*	Oriental fire-bellied toad	Boulenger, 1890	1
*Kurixalus bisacculus*	Taylor’s tree frog	Taylor, 1962	1
*Rhacophorus nigropalmatus*	Wallace’s flying frog	Boulenger, 1895	1
*Kurixalus odontotarsus*	Serrate-legged small treefrog	Ye and Fei, 1993	1
*Gastrotheca riobambae*	Andean marsupial tree frog	Fowler, 1913	1
*Dendrobates leucomelas*	Yellow-banded poison dart frog	Steindachner, 1864	1
*Hylarana nigrovittata*	Black-striped frog	Blyth, 1856	1
*Kaloula pulchra*	Banded bullfrog	Gray, 1831	1
Unknown	–	–	2
Caudata			32
*Ambystoma mexicanum*	Axolotl	Shaw and Nodder, 1798	23
*Tylototriton* spp.	–	–	5
*Tylototriton shanjing*	Emperor newt	Nussbaum, Brodie, and Yang, 1995	2
*Ambystoma dumerilii*	Lake Patzcuaro salamander	Dugès, 1870	1
*Salamandra algira*	North African fire salamander	Bedriaga, 1883	1
Unknown			2

**Table 4:** Number and percentage of positive amphibians regarding gastrointestinal endoparasite infections (total *n* = 161; 66 positive and 95 negative)

Kingdom/phylum	Parasite genus/species	Prevalence/host order (%)	Host species
Metazoa/Nematoda	Rhabditida (*Rhabdias* spp./*Strongyloides* spp.)	Total: 31/161 (19.3)	
		Anura: 29/127 (22.8)	*Agalychnis callidryas* (7), *Agalychnis spurelli* (1), *Bufo regularis* (1), *Ceratophrys cranwelli* (1), *Dendrobates* sp. (1), *Dendrobates tinctorius* (4), *Epipedobates anthonyi* (1), *Kaloula pulchra* (1), *Kurixalus bisacculus* (1), *Litoria caerulea* (6), *Megophrys nasuta* (1), *Osornophryne guacamayo* (1), *Ranitomeya imitator* (1)
		Caudata: 1/32 (3.1)	*Ambystoma mexicanum*, metamorphized (1)
		Unknown: 1/2 (50.0)	Unknown (2)
Metazoa/Nematoda	*Amphibiocapillaria* spp./*Neocapillaria* spp.	Total: 12/161 (7.5)	
		Anura: 11/127 (8.7)	*Agalychnis callidryas* (5), *Dendrobates tinctorius* (2), *Litoria caerulea* (3), *Megophrys nasuta* (1)
		Caudata: 1/32 (3.1)	*Tylototriton shanjing* (1)
Metazoa/Nematoda	*Oswaldocruzia* spp.	Total: 7/161 (4.3)	
		Anura: 7/127 (5.5)	*Agalychnis callidryas* (6), *Litoria caerulea* (1)
Metazoa/Nematoda	*Cosmocerca* spp.	Total: 5/161 (3.1)	
		Anura: 5/127 (3.9)	*Ceratophrys cranwelli* (1), *Litoria caerulea* (3), *Oophaga pumilio* (1)
Metazoa/Nematoda	Oxyurids (Pharyngonoidae)	Total: 4/161 (2.5)	
		Anura: 3/127 (2.7)	*Dendrobates tinctorius* (1), *Litoria caerulea* (2)
		Unknown: 1/2 (50.0)	Unknown: 1/2 (50.0)
Metazoa/Nematoda	Spirurids	Total: 2/161 (1.2)	
		Anura: 2/127 (1.6)	*Phyllobates terribilis* (2)
Protozoa/Metamonada	Flagellated protozoans (unspecified)	Total: 14/161 (8.7)	
		Anura: 11/127 (8.7)	*Bombina microdeladigitora* (1), *Ceratophrys cranwelli* (2), *Ceratophrys ornata* (1), *Dendrobates auratus* (2), *Dendrobates tinctorius* (3), *Litoria caerulea* (2)
		Caudata: 2/32 (6.3)	*Ambystoma mexicanum* (1), *Tylototriton shanjing* (1)
		Unknown: 1/2 (50.0)	Unknown (1)
Protozoa	*Blastocystis* spp.	Total: 5/161 (3.1)	
		Anura: 4/127 (3.1)	*Atelopus wampukrum* (1), *Dendrobates* sp. (1), *Dendrobates tinctorius* (1), unknown (1)
		Caudata: 1/32 (3.1)	*Tylototriton shanjing* (1)
Protozoa/Apicomplexa	Unsporulated coccidian oocyst	Total: 1/161 (0.6)	
		Unknown: 1/2 (50.0)	Unkown (1)
Protozoa/Metamonada	*Tritrichomonas* spp.	Total: 1/161 (0.6)	
		Anura: 1/127 (0.8)	*Dendrobates auratus* (1)
Protozoa/Metamonada	*Karotomorpha* spp.	Total: 1/161 (0.6)	
		Anura: 1/127 (0.8)	*Dendrobates auratus* (1)
Protozoa/Apicomplexa	*Cryptosporidium* spp.	Total: 1/161 (0.6)	
		Anura: 1/127 (0.8)	*Agalychnis callidryas* (1)

**Fig. 1:**
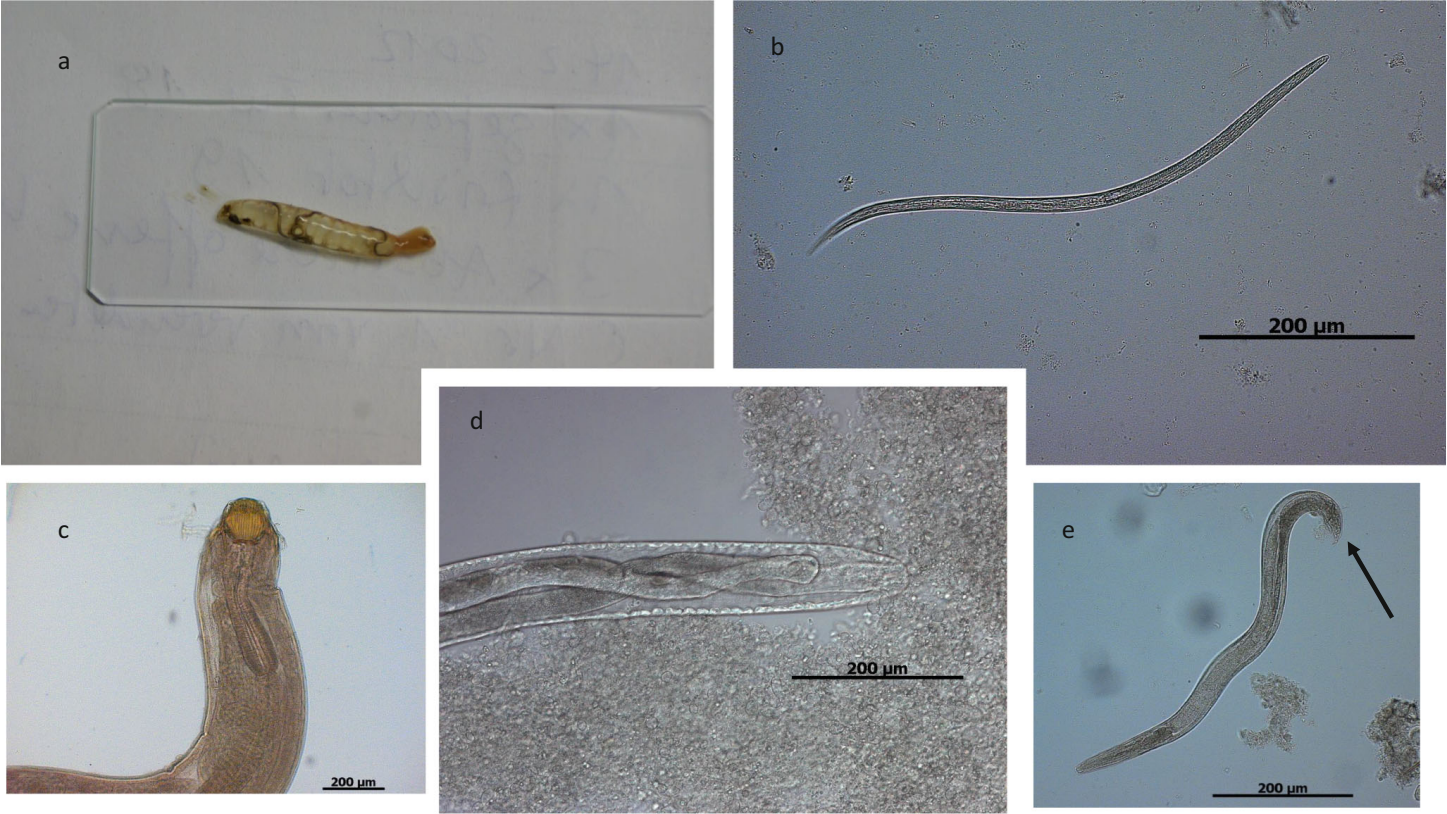
Selected pictures of helminth endoparasites. **a** *Rhabdias* sp.: adult nematodes inside the lung of a red-tailed knobby newt (*Tylototriton kweichowensis*). **b** *Pseudocapillaria* sp.: elongated nematode shed by red-eyed multicolored tree frog (*Agalychnis callidryas*). **c** *Camallanus* sp.: from a Spanish newt, *Pleurodeles waltl.* Please note the anterior buccal capsule armed with teeth. **d** Esophagus of *Capillaria* sp.: shed by an Eastern newt (*Notophthalmus viridescens*). **e** Free-living adult male of *Rhabdias* sp.: please notice posterior end mid gubernaculum, spirules, and cloaca (arrow)

**Fig. 2:**
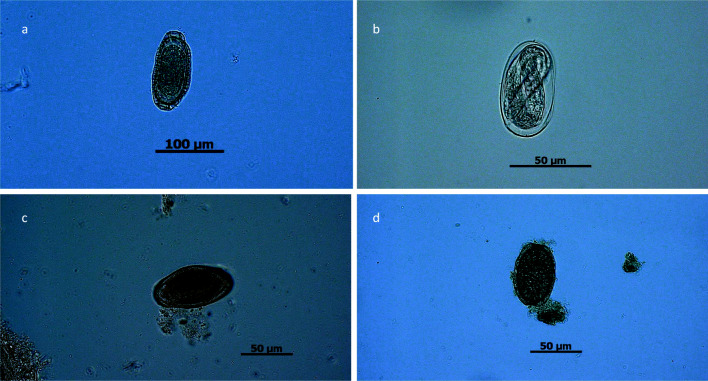
Selected shed stages of endoparasitic nematodes. **a** *Amphibiocapillaria* sp./*Neocapillaria* sp.: brownish lemon-shaped eggs with two pole-clots. Shed by a crocodile newt (*Tylototriton* sp.). **b** Egg of *Rhabdias* sp.: shed by a Marañón Poison frog (*Excidobates mysteriosus*). **c** Oxyurid egg: bean-shaped, thick-walled eggs containing a morula. Shed by an Australian green tree frog (*Litoria caerulea*). **d** *Amphibiocapillaria* sp./*Neocapillaria* sp.: brownish lemon-shaped eggs with two pole-clots. Shed by a white-lipped horned toad (*Megophrys major*)

**Fig. 3:**
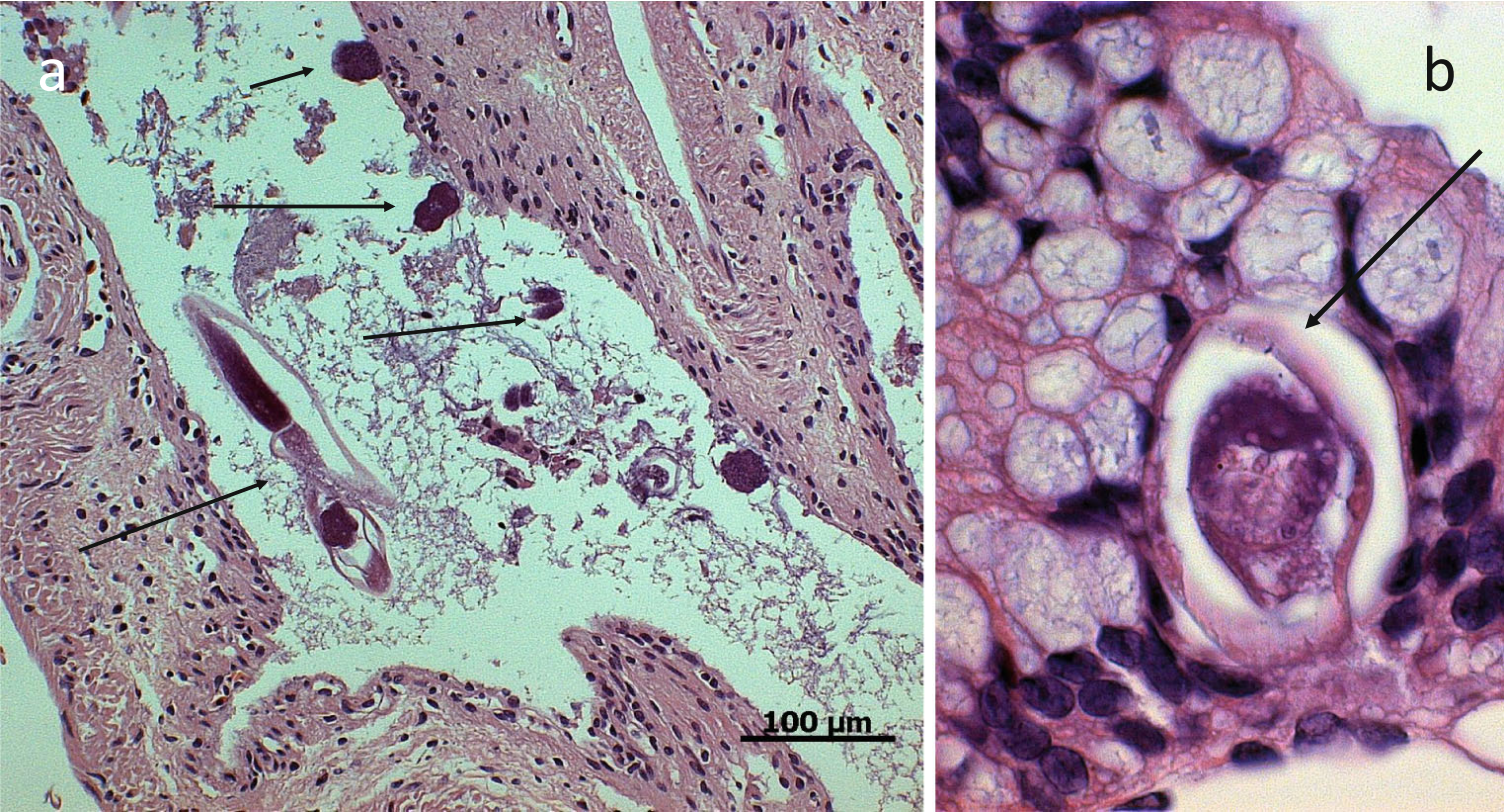
Histology of un-identified nematode infection in a smooth frog (*Theloderma licin*). **a** Notice adult nematode inside the intestinal lumen and diverse site-gated mucosal attached stages (arrows). **b** Enveloped intestinal larval stage: notice thick cuticula of the nematode (arrow)

**Fig. 4:**
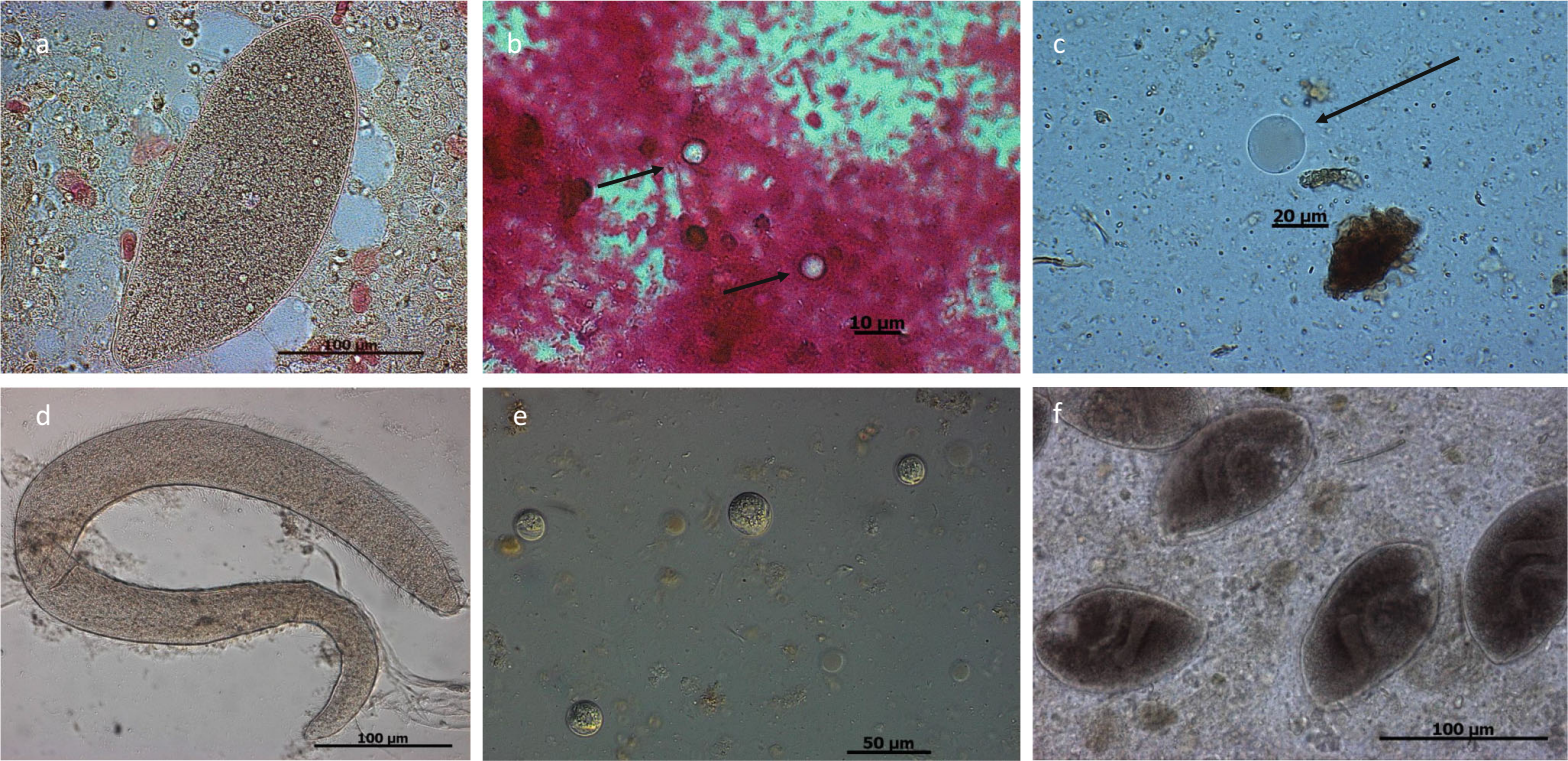
Selected shed stages of protozoan endoparasites/commensals in amphibians. **a** *Opalina* sp.: heterokont from a yellow-bellied toad (*Bombina variegata*). Sparozoic *Opalina* lacking a mouth (cytostome) and covered with flagelliformic cilia. Inside are numerous similar nuclei. **b** Carbolfuchsin-stained fecal smear. Clearly detached are shed oocysts of *Cryptosporidium* sp. (arrows) by an Australian green tree frog (*Litoria caerulea*). These oocysts might come from prey animals, since captive Australian green tree frogs were fed with baby mice (Mutschmann, personal communication). **c** Vacular form of *Blastocystis* sp.: shed by Cranwells horned frog (*Ceratophrys cranwelli*). **d** *Protoopalina* sp.: heterokont form *Hyperolius* sp., *Protoopalina* sp., such as *Opalina* sp., seem to be most likely commensal, than parasitic. **e** Spores of *Basidobolus* sp.: *Basidobolus* is a filamentous fungus known to cause zygomycosis in amphibians, and shed spores can easily be mistaken for un-sporulated coccidian oocysts. **f** Trophozoites of *Nyctotherus* sp.: large trophozoites with lateral cytostomes and prominent macronuclei. Shed by red-eyed multicolored tree frog (*Agalychnis callidryas*)

Illustrations of selected parasitic stages and histopathological findings are shown in Figs. 1, 2, 3, and 4. Applying CFS staining (Heine 1982), one Australian green tree frog (*Litoria caerulea*) out of 73 analyzed samples was positive for *Cryptosporidium* oocysts (see Fig. 4b). According to taxonomic order, parasite infection rates of anuran and caudata amphibians differed significantly (Chi-square test: *χ*^2^ = 7.7, df = 1, *P* = 0.01; *r* = 0.27; 95% CI [0.07–0.46]), being higher in frogs/toads (51.12%) than in salamanders (12.88%). In addition, *Rhabdias/Strongyloides* infection rates varied within taxon (Fisher’s exact test: *P* = 0.01; *r* = 0.44; 95% CI [0.03–0.72]), as such that caudates were less frequently infected (3.13%) than anurans (22.83%). For other detected parasite species, no significant levels were observed within different amphibian hosts. Furthermore, no significant correlation in parasitic burdens was detected in relation to other analyzed factors, such as keeping facility (zoo, private household), age, sex, group size, and maintenance conditions. Finally, there were no significant differences of parasitic infection rates when comparing alive from deceased amphibians.

In total, 42 dissected amphibians out of 107 (39.3%) were positive for endoparasite infections (Table 5). Twenty amphibians (18.7%) died due to severe protozoan- and metazoan-induced enteritis: *Spironucleus*, *Tritrichomonas*, ciliate infections (3.7%), as well as cosmocercosis (2.8%), amphibiocapillariosis/neocapillariosis (1.9%), amebosis (1.9%), rhabdiosis (0.9%), strongyloidiosis (0.9%), rhigonemosis (0.9%), aplectanosis (0.9%), nematotaeniosis (0.9%), and mesomycetososis (0.9%). Other etiological causes identified for amphibian deaths included bacterial infections [*Chryseobacterium indologenes*, *Citrobacter freudii*, *Sphingobacterium multivorum*, *Klebsiella pneumoniae*, *Aeromonas hydrophila*, *Pseudomonas fluorescens/P. luteola*, *Stenotrophomonas maltophila*, *Elizabethkingia* spp., and *Serratia* spp. (33 animals, 30.8%), mycobacteriosis (4 animals, 3.7%), brucellosis (1 animal, 0.9%) and clamydiosis (one animal, 0.9%)] and/or fungal infections [chytridiomycosis (19 animals, 17.8%), *Candida* spp., *Mucor amphibiorum*, *Cladosporium* spp., *Basidobolus* spp., *Saprolegnia* spp., and chromomycosis (12 animals, 11.2%)]. Concerning non-infectious death causes, we identified in a Betsileo Madagascar frog (*Mantidactylus betsileanus*) a severe egg-related stasis most probably due to reproductive disorders. Moreover, in a Dyeing dart frog (*Dendroabtes tinctorius*), we found an advanced renal carcinoma, and in an African clawed frog (*Xenopus laevis*), a cutaneous lymphadenoma. In 14 (13.1%) animals, the final cause of death remained unknown. Besides, one *Batrachochytrium dendrobatidis*-infected axolotl salamander (*Ambystoma mexicanum*) was co-infected with *Ichthyobodo* spp. ectoparasites*.* An overview on dissected animals, the diagnosed endoparasites and combined microbiological, pathological, and pathohistological findings is provided in Table 6.

**Table 5:** Number and percentage of positive amphibian corpses regarding gastrointestinal endoparasite infections (total *n* = 107; 42 positive and 14 different gastrointestinal parasites detected)

Kingdom/phylum	Parasite species	Prevalence (%)	Host species (*n*)
Metazoa/Nematoda	*Amphibiocapillaria* spp./*Neocapillaria* spp.	Total: 8/107 (7.5)	
		Anura: 6/64 (9.4)	*Dendrobates tinctorius* (1), *Hylarana cubitalis* (1), *Kurixalus bisacculus* (1), *Litoria caerulea* (1), *Rhinella marina* (1), *Xenopus laevis* (1)
		Caudata: 2/42 (4.8)	*Notophthalmus viridescens* (1), *Triturus pygmaeus* (1)
Metazoa/Nematoda	*Cosmocerca* spp.	Total: 8/107 (7.5)	
		Anura: 5/64 (7.8)	*Bombina bombina* (1), *Dendrobates* sp. (1), *Hylarana cubitalis* (1), *Litoria caerulea* (1), *Mantidactylus betsileanus* (1)
		Caudata: 3/42 (7.1)	*Salamandra algira splendens* (2), *Salamandra salamandra* (1)
Metazoa/Nematoda	Rhabditida (*Rhabdias* spp./*Strongyloides* spp.)	Total: 3/107 (2.8)	
		Anura: 2/64 (3.1)	*Dendrobates auratus* (1), *Litoria caerulea* (1)
		Caudata: 1/42 (2.4)	*Salamandra crexpoi* (1)
Metazoa/Nematoda	*Oswaldocruzia* spp.	Total: 3/107 (2.8)	
		Anura: 3/64 (4.7)	*Bombina variegata* (1), *Dendrobates tinctorius* (2)
Metazoa/Nematoda	*Aplectana* spp.	Total: 3/107 (2.8)	
		Anura: 2/64 (3.1)	*Dendrobates auratus* (1), *Hylarana cubitalis* (1)
		Caudata: 1/42 (2.4)	*Salamandra salamandra* (1)
Metazoa/Platyhelminthes	Cestodes (unspecified)	Total: 2/107 (2.8)	
		Anura: 3/64 (4.7)	*Bombina variegata* (1), *Dendrobates tinctorius* (1), *Hylarana cubitalis* (1)
Metazoa/Trematodes	Trematode eggs (Digenea)	Total: 1/107 (1.4)	
		Anura: 1/64 (1.6)	*Bombina variegata* (1)
Protozoa/Metamonada	*Trichomonas* spp.		
		Anura: 6/64 (9.3)	*Dendrobates tinctorius* (1), *Oophaga histrionica* (1), *Phyllobates terribilis* (1), *Theloderma corticale* (3)
		Caudata: 1/42 (0.2)	*Ambystoma tigrinum* (1)
Protozoa/Metamonada	Flagellated protozoa (unspecified)	Total: 5/107 (4.7)	
		Anura: 2/64 (3.1)	*Phyllobates bicolor* (1), *Ranitomeya imitator* (1)
		Caudata: 3/42 (7.1)	*Cynops pyrrhogaster* (1), *Triturus pygmaeus* (1), *Tylototriton* sp. (1)
Protozoa/Metamonada	*Spironucleus* spp.	Total: 5/107 (4.7)	
		Anura: 1/64 (1.6)	*Gastrotheca riobambae* (1)
		Caudata: 4/42 (9.5)	*Ambystoma tigrinum* (1), *Cynops pyrrhogaster* (1), *Salamandra crexpoi* (1), *Salamandra salamandra* (1)
Protozoa	*Entamoeba* spp.	Total: 3/107 (2.8)	
		Anura: 3/64 (4.7)	*Atelopus hoogmoedi* (1), *Litoria caerulea* (1), *Oophaga histrionica* (1)
Protozoa	*Neobalantidium* spp.	Total: 3/107 (2.8)	
		Anura: 3/64 (4.7)	*Theloderma corticale* (1), *Xenopus laevis* (2)
Protozoa/Metamonada	*Tritrichomonas* spp.	Total: 3/107 (2.8) Caudata: 3/42 (7.1)	
		Caudata: 3/42 (7.1)	*Salamandra crexpoi* (1), *Salamandra salamandra* (1), *Tylototriton sp.* (1)
Protozoa/Metamonada	*Karotomorpha* spp.	Total: 3/107 (2.8)	
		Anura: 3/64 (4.7)	*Oophaga histrionica* (1), *Theloderma corticale* (2)
Protozoa	*Nyctotherus* spp.	Total: 2/107 (1.9)	
		Anura: 2/64 (3.1)	*Xenopus laevis* (2)
Protozoa/Apicomplexa	*Eimeria* sp.	Total: 1/107 (0.9)	
		Caudata: 1/42 (0.2)	*Salamandra salamandra* (1)

**Table 6:** Performed necropsies of amphibians regarding infection with endoparasites, microbiological results, and pathological/pathohistological findings

Amphibian host	Parasites detected	Microbiology (liver/coeloma)	Pathohistological findings/pathological findings
*Tylototriton* sp.	*Spironucleus* sp. ++/*Tritriomonas* sp. ++	–	Hemorrhagic-necrotizing enteritis
*Dendrobates auratus*	*Strongyloides* sp. ++/*Aplectana* sp. ++	–	Systemic chromomycosis
*Litoria caerulea*	*Rhabdias* sp. +++	*Chryseobacterium indologenes* +++, *Pseudomonas fluorescens* +	Hepatitis, nephritis, nematode-eggs in faveoli, mild pneumonia
*Cynops pyrrhogaster*	Unspecified flagellates (++), *Spironucleus* sp. (++)	*Aeromonas hydrophila* +++, *P. fluorescens* +++, *Sphinogbacterium multivorum* ++	Catharalic enteritis, hepatitis, nephritis, bacterial sepsis
*Hylarana cubitalis*	Oxyuridae (+)/*Aplectana* sp. +	–	Autolytic
*Salamandra crexpoi*	*Strongyloides* sp. ++/S*pironucleus* sp. +++	*A. hydrophila* +++, *P. fluorescens* +++, *C. indologenes* ++	Catharalic enteritis, bacterial liver infection
*Dendrobates tinctorius*	Cestode eggs (*Nematotaenia* sp.) ++	–	Obstipation in the large intestine with adult cestodes and ground substrate, Ziel-Neelsen staining negative
*Xenopus laevis*	*Pseudocappillaroides xenopi* ++ (skin)/*Neobalantidium* sp. ++ (apathogen), Opalinids + (apathogen)	*A. hydrophila* ++/*P. fluorescens* +++/*C. indologenes* ++	Necrotizing dermatitis, hemorrhagic-necrotizing enteritis, liver and kidney degeneration
*Mantella aurantiaca*	*Rhigonema ingens* +++	*Enterobacter faecalis* ++	Cachectic, vaculous liver degeneration, kathr-hermorrhagic enteritis
*Ranitomeya imitator*	Unspec. flagellates (++)	–	–
*Mantidactylus betsileanus*	*Cosmocerca* sp. ++	–	Ovulatory eggbound
*Litoria caerulea*	*Amphibiocapillaria* sp. +++	*A. hydrophila* ++/*C. indologenes* ++	–
*Salamandra algira splendens*	*Cosmocerca* sp. ++	*Mucor amphibiorum +++*	*Mucor amphibiorum* infection
*Triturus pygmaeus*	*Amphibiocapillaria* sp. +++/Unspec. flagellates (++)	*Citrobacter freundii* ++/*A. hydrophila* ++	Edema in the central nervous system
*Salamandra algira splendens*	*Cosmocerca ornata* ++	–	Mycotic profound dermatitis, mycotic granuloma in the liver (knods), Dematiaceae infection
*Rhinella marina*	*Amphibiocapillaria* sp. +++	–	*Batrachochytrium dendrobatidis* infection (Chytridiomycosis)
*Theloderma corticale*	*Tritriomonas* sp. ++	*A. hydrophila* ++/*C. indologenes* ++	–
*Theloderma corticale*	*Karotomorpha* sp. +++/*Tritriomonas* sp. ++	*A. hydrophila* +++/*P. fluorescens* +++	Enteritis, hepatitis
*Theloderma corticale*	*Tritriomonas* sp. ++/*Karotomorpha* sp. +++	*A. hydrophila* +++/*P. fluorescens* +++	Enteritis, hepatitis
*Hylarana cubitalis*	Cestode eggs +/*Cosmocerca* sp. ++/*Amphibiocapillaria* sp. +++	–	–
*Kurixalus bisacculus*	*Amphibiocapillaria* sp. +++	*C. braakii* +++/*P. fluorescens* +++	Serositis, nephritis, abscesses in liver, hepatitis, adnexitis
*Gastrotheca riobambae*	*Spironucleus* sp. +++	*C. freundii* +++/*P. fluorescens* +++	Pneumonia, inflammation of the liver and the gut
*Dendrobates tinctorius*	*Oswaldocruzia* sp. ++/*Tritriomonas* sp. ++	–	*Batrachochytrium dendrobatidis* infection (Chytridiomycosis)
*Salamandra salamandra*	*Eimeria* sp. +++/*Aplectana* sp. +++/*Spironucleus* sp. +/*Tritriomonas* sp. +++	–	Chronic enteritis, mycotic dermatitis (Dematiaceae)
*Theloderma corticale*	*Neobalantidium* sp. +++	*P. fluorescens* ++/*Stenotrophomonas maltophila* +	Generalized edema, enteritis, hepatitis, bacterial infection
*Dendrobates* sp.	*Cosmocerca* sp. +++	–	Enteritis, cosmocercosis
*Ambystoma tigrinum*	*Tritriomonas* sp. +/S*pironucleus* sp. +++	*P. fluorescens* ++/*C. indologenes* +++	Enteritis, granulomatous hepatitis
*Bombina variegata*	*Oswaldocruzia* sp. ++/Cestode eggs ++	*P. fluorescens* +++	Generalized edema, enteritis, potentially intoxication
*Oophaga histrionica*	*Karotomorpha* sp. +++/*Tritriomonas* sp. +++/*Entamoeba* sp. +++	*P. fluorescens* +++ *C. braakii* +++/*Elisabethkingia* sp. ++ (skin)	Enteritis, profound dermatitis (head, back)
*Dendrobates tinctorius*	*Amphibiocapillaria* sp. +/*Physoloptera*-like nematodes +++	–	Anemia, dermatitis
*Notophthalmus viridescens*	*Neoocapillaria* sp. +	–	Hemorrhagic-necrotizing enteritis, anemia
*Phyllobates terribilis*	*Tritriomonas* sp. +++	*C. freundii* ++/*A. hydrophila* +++	Bacterial inflammation of the lungs, the kidney, and the liver
*Bombina bombina*	*Cosmocerca* sp. +++	*Citrobacter* sp. ++/*Klebsiella* sp. +/*Providencia rettgeri* +++	Cachexia, hydrocoeloma, enteritis, granulomatous hepatitis, chronic nephritis
*Litoria caerulea*	*Cosmocerca* sp. +++	–	Calcified nematode stages, anemia, cachexia, enteritis, lymphoma
*Bombina variegata*	Trematode eggs (Digenea) +++	*Citrobacter* sp. +	Hydrocoeloma, generalized edema, hepatitis, enteritis, nephritis, bacterial infection
*Xenopus laevis*	*Pseudocappillaroides xenopi* ++ (skin)/*Neobalantidium* sp. +/*Nyctotherus* sp. (apathogen) ++	*C. freundii* ++ (liver, skin)/*A. hydrophila* +++ (liver/skin)	Parasitic dermatitis, microgranuloma with central necrosis in the liver, kidney congestion, bacterial secondary infection of the skin
*Salamandra salamandra*	*Cosmocerca ornata* ++	*Citrobacter braakii* ++/*Chryseobacterium indologenes* +++/*P. fluorescens* + (gut)	Hepatitis, enteritis
*Dendrobates tinctorius*	*Oswaldocruzia* sp. ++	–	*Batrachochytrium dendrobatidis* infection (Chytridiomycosis), catharalic enteritis

## Discussion

Parasite infections in free-ranging amphibians seem to appear obligatory worldwide, and thus, very high prevalences of up to 90% have previously been described (Coggins and Sajdak 1982; Al-Sorakhy and Amr 2003; Amin et al. 2012). For instance, Rizvi et al. (2011) sampled free-ranging amphibians in an Indian Wildlife Sanctuary (Haryana) and found that endemic common dicroglossid frogs (*Euphlyctis cyanophlyctis*) were frequently infected (52.9%) by nematodes. In contrast to this wildlife study, there is very little knowledge on parasitic infections of dicroglossid frogs (*E. cyanophlyctis*) kept in captivity. While comparing our prevalence data with previous published studies, it should be considered that most of these surveys were conducted in wild animals, and this fact might explain prevalence differences. Most likely to dicroglossid frogs, other free-ranging amphibians are also showing higher parasitic prevalences when compared with those kept in captivity (Coggins and Sajdak 1982; Amin et al. 2012). Moreover, sensitivity and specificity of applied DSFS to detect helminth and protozoan stages might have influenced observed prevalence as different diagnostic methods in former wildlife studies have been used (Rizvi et al. 2011; Amin et al. 2012).

Despite the fact that extrinsic risk factors, such as habitat changes, habitat losses, predatory pressure, and poor water quality can directly affect parasitic burdens and prevalences in free-ranging amphibians (Vaucher 1990; Kehr and Hamann 2003; Marcogliese and Pietrock 2011; Thiemann and Wassersug 2000), very little is still known whether these factors might also influence the outcome of parasitic burdens in pet amphibians kept in households or zoos (Mutschmann 2010).

In this study, helminth infections occurred frequently in investigated animals (Table 4). All nematode species found in this survey have been reported to possess pathogenic significance for amphibians (Mutschmann 2010; Amin et al. 2012; Langford and Janovy 2009; Langford 2010; Yildirimhan et al. 2012). Correspondingly, amphibians are well-known to be parasitized by numerous nematode families, such as Trichinellidae, Rhabditidae, Strongyloididae, Ascarididae, Cosmocercodidae, Oxyuridae, Heterakidae, Camalladae, Gnathostomatidae, Habronematidae, Filaroidae and Physalopteridae. For amphibians, particularly rhabditidean helminths are considered as pathogenic endoparasites (Mutschmann 2010; Amin et al. 2012; Yildirimhan et al. 2012). The genus *Strongyloides* is known to cause protein-loss enteropathy in various anuran hosts (Patterson-Kane et al. 2001). Cosmopolitan adult female *Rhabdias* lungworms are capable of parthenogenesis and known to parasitize lung tissues of different amphibian hosts, including various toad and frog species (Langford 2010; Fernández Loras et al. 2011), while males live in earth/ground substrates (geohelminths). Amphibian hosts become infected by oral uptake or percutaneous infection of exogenous infective third-stage larvae (L3) which then migrate via blood/lymph system into the lungs (Langford and Janovy 2009; Langford 2010). In lungs, adult *Rhabdias* females start producing eggs through parthenogenesis. Thus, amphibian rhabdiosis might result in pulmonary tissue damage and/or eosinophilic pneumonia (Densmore and Green 2007). In free-ranging amphibians, *Rhabdias* infections seem to occur frequently and sometimes result in pneumonia (Kuzmin et al. 2003; Mohammad et al. 2010; Fernández Loras et al. 2011). Consistently, *Rhabdias* spp. infection rates for captive German amphibians were rather high in this study (19.3%) and resulted in the most prevalent parasites. *Rhabdias/Strongyloides* infection rates varied significantly within taxon, i.e., caudates were less frequently infected (3.13%) than anurans (22.83%). Nonetheless, it is well known from literature that *Rhabdias* is more frequently parasitizing frogs/toads (Langford and Janovy 2009; Langford 2010). In line, *Rhabdias ranae* seems not capable to infect caudates and to be restricted to frogs/toads as suitable hosts, but in the past two decades, first *Rhabdias* infections in caudates have been reported (Kuzmin et al. 2001; Kuzmin et al. 2003; Eisenberg and Pantchev 2009). Therefore, it seems assumable that anurans might be more often infected with *Rhabdias* than caudates, especially because the correlation was rather high (*r* = 0.44) when comparing these two amphibian groups (Cohen 1988). Clinical relevance of rhabdiosis was also underlined in dissections, since in one adult male Australian green tree frog (*L. caerulea*), a *Rhabdias* spp.-infected lung was found and which might have caused severe pneumonia, hepatitis, and nephritis. Nonetheless, other pathogens could not be ruled out as the same animal showed secondary bacterial infections with *Chryseobacterium indologenes* (+++) and *Pseudomonas fluorescens* (+) isolated from the frog’s coeloma. Alongside *Rhabdias*, other nematode genus, i.e., *Oswaldocruzia*, was frequently diagnosed (2.8%) in domestic kept amphibian pets. *Oswaldocruzia* nematodes infect amphibian hosts exclusively by the oral uptake of exogenous infective L3 (Hendrikx 1983). Noteworthy, a cutaneous *Pseudocapillaroides xenopi* infection was diagnosed in an adult African clawed frog (*X. laevis*). This *X. laevis*-infected animal suffered not only of a severe verminous dermatitis but also of secondary Gram-negative *P. fluorescens* (+++), *Aeromonas hydrophila* (++), and *Citrobacter braakii* (++) dermal infections. The amphibian nematode *P. xenopi* infects the epidermis and can cause clinically symptoms, such as erythematous/erosive dermatitis, with characteristic roughness of affected skin, petechiae, and dermal ulcera (Cunningham et al. 1996; Mutschmann 2010). *P. xenopi* can complete its direct life cycle within epidermis of frogs/toads in which burrowing activities of subdermal nematodes can lead to the damage of parasitized skin. Therefore, *P. xenopi*-infected animals are more susceptible for bacterial and/or fungal secondary dermal infections (Cunningham et al. 1996), as confirmed in our investigation.

According to protozoan enteric infections, in 14 cases (8.7%), potentially pathogenic, flagellated protozoan genera, such as *Proteromonadida*, *Reteromonadida*, *Diplomonadida*, and *Trichomonadida*, were additionally diagnosed. Nonetheless, the literature considers many of these enteric flagellates as commensals within intestinal tract of amphibians (Densmore and Green 2007; Mutschmann 2010). Conversely, some genera of diplomonadids (*Giardia*, *Hexamita*, *Spironucleus*) and trichomonadids (*Monocercomonas*, *Hexamastix*, *Tritrichomonas*) can cause weight loss, general edema, and enteritis in severely infected animals.

The clinical relevance of flagellated protozoan infections was demonstrated during conducted dissections: Out of all dissected animals, four (3.7%) died because of severe *Tritrichomonas* spp.- and/or *Spironucleus* spp.-derived enteritis. These animals showed severe catarrhalic- to hemorrhagic-necrotic enteritis combined with secondary bacterial infections (e.g., *Pseudomonas* spp./*Sphingobacterium* spp.) of liver and gut mucosa.

Only five animals (3.7%) were positive for *Blastocystis* spp. infections. Conversely to our findings, Yoshikawa et al. (2004) found anurans and newts from distinct locations in Japan to be infected with *Blastocystis* showing very high prevalences (47.8–100%) by using in vitro culture diagnostic methods. Our observed *Blastocystis* prevalence might have been higher if this in vitro cultivation method would have been applied, but it cannot be excluded that this parasite is simply less frequently found in German pet amphibians. Unfortunately, there is still very little knowledge on amphibian-related blastocystiosis. The same holds true for its possible impact on animal health kept in captivity (Mutschmann 2010). Nevertheless, *Blastocystis* should be considered as potentially pathogenetic protozoan species and infections should be considered according to clinical symptoms. Moreover, during dissections we here diagnosed *Entamoeba* spp. cysts in three (2.8%) animals.

Several studies have focused on gastrointestinal apicomplexan coccidian parasites in amphibians (Duszynski et al. 2007). So far, monoxenous coccidian genera *Eimeria*, *Goussia*, *Hyaloklossia*, and *Cystoisospora* (former *Isospora* according to new nomenclature) have been described in diverse amphibian host species (Duszynski et al. 2007), and for further review a disposed online version (http://biology.unm.edu/coccidia/anura.html) is recommended. In accordance with these reports, we also diagnosed un-sporulated coccidian oocysts in one animal (0.6%), but amphibian oocysts were not fully identifiable to species level. Furthermore, non-sporulated *Eimeria* spp. oocysts were found within gut lumen of one dissected fire salamander, (*Salamandra salamandra*) but coccidian-derived death was ruled out as this animal was also co-infected with *Aplectana* spp., *Spironucleus* spp., and *Tritrichomonas* sp. and showed a manifested mycotic dermatitis.

Enteropathogenic apicomplexan *Cryptosporidium* is known to infect also the microvillus border of amphibian gastrointestinal epithelial cells (Jirků et al. 2008). Consistently, we diagnosed *Cryptosporidium* oocysts in an Australian frog (*L. caerulea*) via CFS analysis. If here identified *Cryptosporidium* oocysts were shed during a patent infection or whether they were passed because of *Cryptosporidium* spp.-infected prey animal consumption (e.g., feeding of baby mice) remains unclear. Since *Cryptosporidium* can be transmitted by ingestion of infected food animals, poorly treated water as well as direct contact with infective oocysts, it is possible to assume that human infections might occur through ingestion of under-cooked frog (*Rana* spp.) meat and/or handling and processing of *Cryptosporidium*-infected frogs as recently demonstrated in Africa (Kia et al. 2017). Former study revealed a high prevalence of *Cryptosporidium* spp. (35.9%) in the intestine of 117 frogs (*Rana* spp.) sold at the Hanwa frog market Zaria, Kaduna State, Nigeria, for human consumption (Kia et al. 2017; Kia and Ukuma 2017). Therefore, further public health studies on different transmission routes of this neglected anthropozoonotic parasite should be conducted, including amphibians designated for human consumption (Kia et al. 2017; Kia and Ukuma 2017).

Aside from protozoans, nematodes, cestodes, and trematodes, no acanthocephalan infections were here detected. Nonetheless, during necropsies, also cestode-parasitized animals were found. As such, in three dissected animals (2.8%), various long cestode specimens containing mature proglottids were diagnosed. Noteworthy was a heavily *Nematotaenia*-infected male Dyeing dart frog (*Dendrobates tinctorius*), which showed obstipation and congestion of ground substrate in the gut lumen. Amphibians are known to be infected by different cestode genera, i.e., *Proteocephalus*, *Ophiotaenia*, *Cephalochlamys*, *Bothriocephalus*, *Nematotaenia*, *Distoichometra*, *Cylindrotaenia*, and *Baerietta*. Clinical symptoms of nematotaeniosis manifest in affected animals during stress and/or in case of heavy infections (Mutschmann 2010). Then, ileus with obstipation, blood loss, necrosis of intestinal mucosa, edema or even death may also occur if untreated (Mutschmann 2010). Interestingly, a digenean trematode infection was found in a deceased yellow-bellied toad (*Bombina variegata*), showing clinical symptoms, including hydrocoeloma, generalized edema, and pathohistological findings, such as hepatitis, enteritis, nephritis, and a bacterial co-infection (*Citrobacter* spp. +). Amphibians represent not only intermediate hosts for various digenean trematode orders (e.g., Amphistomida, Echinostomatida, Gasterostomida, Hemiurida, Holostomida, Plagiorchida) but also second or even final hosts. Nevertheless, trematode-driven pathological effects are mostly unknown for amphibians (Mutschmann 2010).

Since many of examined amphibians in this study are considered as threatened endemic species of neotropical regions, e.g., *Adelphobates galactonotus*, *Phyllomedusa bicolor*, and *Trachycephalus resinifictrix*, and thus being kept as zoo animals for conversation reasons, detected parasites in these animals might represent imported parasites from their natural tropical habitats. Therefore, it seems noteworthy to mention that new wild amphibians introduced into zoological gardens should undergo a mandatory quarantine regime in order to avoid further spread of neozoan parasites as suggested elsewhere (Hallinger et al.^ 2019^; ^2020^).

Interestingly, parasitic infection rates in investigated anurans (51.12%) were significantly higher than the ones observed in caudate species (12.88%). As proposed for zoo animals, it is also recommended that newly purchased frogs, newts, and toads by private owners should be submitted to parasitological examination in order to detect presence of gastrointestinal parasites during quarantine as a routine health screening.

## Conclusions

Our representative epidemiological survey on endogenous parasites of captive amphibians in Germany found several pathogenic parasite species resulting in clinically manifested disease. If correctly diagnosed, identified parasitoses should be medicated taking into account commensalism, metabolic features of amphibians, clinical signs, and more importantly prophylactic approaches in order to prevent future infections. Applied DSFS technique on scat samples proved to be valid for detection of many relevant parasitic stages, including tiny protozoan oocysts. Since biology, epidemiology, as well as pathogenesis of most amphibian parasitoses are not well understood, further investigations in these directions are needed. Similarly, the current lack of suitable therapy options for many of these amphibian parasitoses calls for more research in new drug development within the field of neglected herpetology medicine.
